# Supplementary material for: Outcome after acute ischemic stroke is linked to sex-specific lesion patterns
Source: Nat Commun. 2021 Jun 2;12:3289. doi: 10.1038/s41467-021-23492-3 (PMC8172535; doi:10.1038/s41467-021-23492-3)
Supplement: Supplementary file 1 — Supplementary Information [file 41467_2021_23492_MOESM1_ESM.pdf]

Supplementary Information

Supplementary note 1: Derivation cohort: GASROS

Sample size derivation

We had access to 668 patients with manual lesion segmentations. Quality control of normalization results of structural images led to the exclusion of 55 out of these 668 patients. Included and excluded patients did not differ significantly with respect to age, sex and stroke severity (mean age(SD): 65.0(15.1) vs. 64.0(14.8), p=0.66, sex: 38% female vs. 35% female, p=0.77, mean NIHSS(SD): 5.04(6.0) vs. 6.1(6.0), p=0.24). 555 out of the 613 remaining patients had complete data with respect to clinical variables (i.e., age, sex, stroke severity, comorbidities, WMH lesion volume) and were thus included in final analyses.

Unsupervised lesion embedding derivation

We employed non-negative matrix factorization (NMF) to compute a low-rank approximation of our lesion data: Let the matrix **V** represent the region-wise lesion loads for all patients and thus be of p x n dimensions (p = number of grey matter regions/white matter tracts and n = number of patients). NMF decomposes the matrix **V** into a latent factor representation **W** and a latent factor loading **H**, i.e., **V**=**WH** with p x k and k x n dimensions (k=number of latent spatial patterns).[1] The hidden factor representation **W** then links the derived low-rank lesion embedding to the lesion information in the original anatomical brain regions. The matrix of hidden factor loadings **H** assigns relevances to each specific lesion pattern within the lesion embedding to characterize individual patients' actual lesions. We opted for this multi-to-multi mapping representation, as we expected to derive biologically more meaningful stroke patterns in view of NMF's positivity constraint. This important quality of NMF stands in contrasts to alternative matrix factorizations algorithms, such as principal component analysis (PCA): When using this alternative decomposition approach, an individual patient's lesion would have been retrieved through inscrutable additions and subtractions of low-dimensional lesion pattern, which would have hindered an intuitive and direct interpretation of lesion pattern effects.

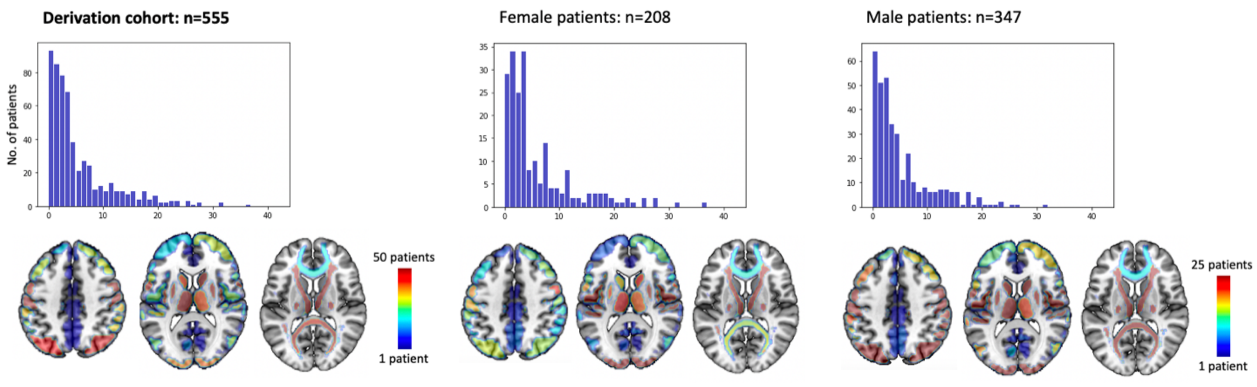

Supplementary Figure 1. NIHSS distributions and frequency maps for the entire derivation cohort, as well as subgroups of only female and male patients. Source data are provided as a Source Data file.

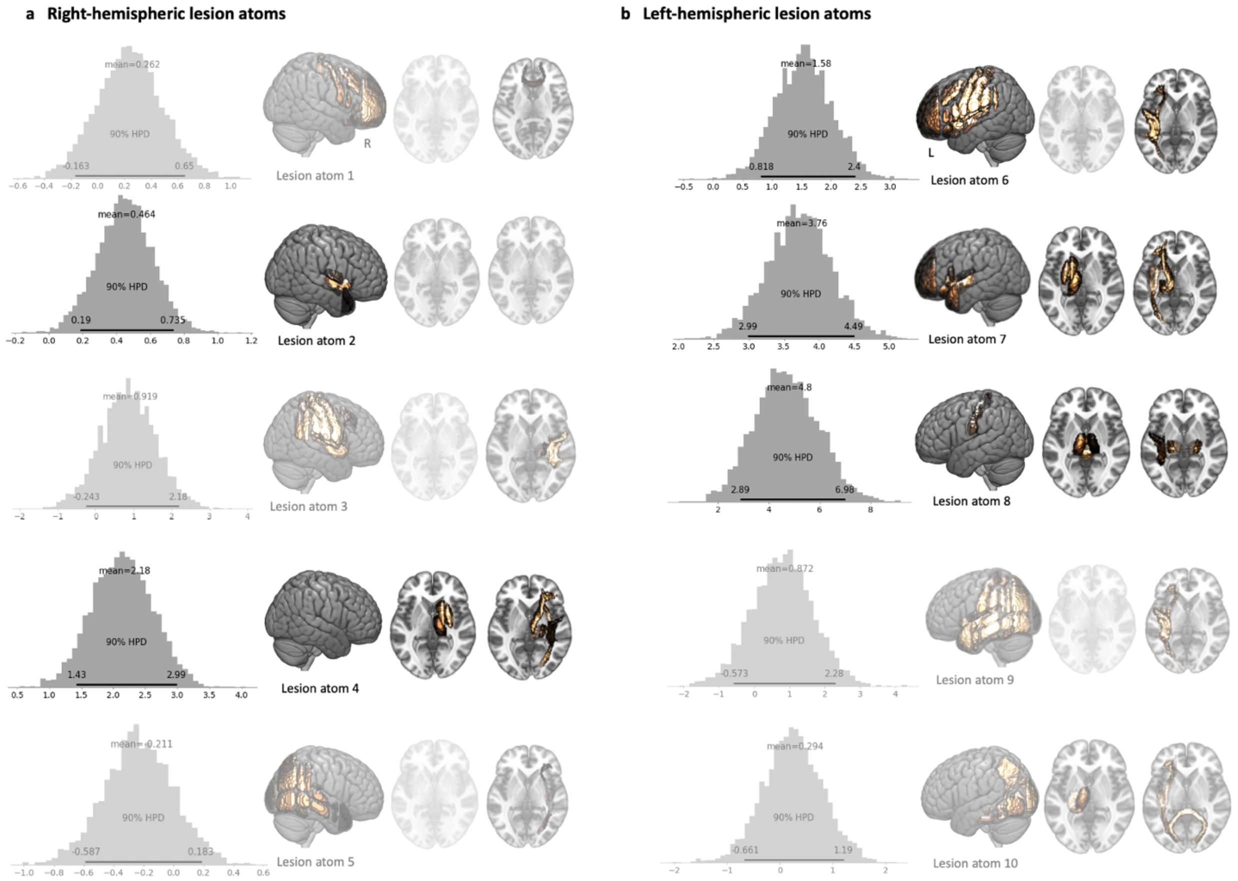

Supplementary Figure 2. Bayesian posterior distributions of lesion atoms in the right (a) and left (b) hemisphere that substantially diverged from zero. Right-hemispheric lesion atom 4 and left-hemispheric lesion atoms 7 and 8 presented the highest predictive relevances of stroke

severity. Bayesian posterior distributions of those lesion atoms that did not substantially diverge for zero are shown in transparent. Source data are provided as a Source Data file.

Supplementary note 2: Validation cohort: MRI-GENIE

AIS patients in the validation dataset originated from the MRI-GENIE study.[2] Out of 2,765 automatically segmented lesions,[3] 1,920 (70.1%) passed internal quality control by two raters (M.B., A.K.B.). Included and excluded patients did not differ with respect to age, sex, NIHSS stroke severity and Rankin Scale-based functional outcome (p>0.05, Bonferroni-corrected for four comparisons). DWI-defined lesions were spatially normalized to MNI-space.[4] The volume of white matter hyperintensity lesions was obtained via a previously developed, fully automated deep learning-based segmentation pipeline of the white matter hyperintensities on FLAIR images.[5]

Initial NIHSS-based stroke severity was available for 942 MRI-Genie patients from six international centers. We excluded those patients that were enrolled in the GASROS study to prevent an overlap of data between the derivation and validation cohort (n=150). Automatically segmented lesion outlines were available for 503 out of the remaining 792 patients with information on stroke severity. Thus, these 503 patients (mean age(SD): 65.0 (14.6), sex: 40.6% female, mean NIHSS(SD): 5.48 (5.35)) originating from five centers constituted the finally included sample (c.f., **Supplementary Table 1** for further clinical characteristics). Patients gave written informed consent in accordance with the Declaration of Helsinki. The study protocol was approved by Massachusetts General Hospital's Institutional Review Board (Protocol #: 2001P001186 and 2003P000836).

Supplementary Table 1. Patient characteristics of the validation cohort. Mean (SD) unless otherwise noted. The groups of male and female patients were compared via two-sample t-tests or two-sided Fisher's exact tests as appropriate. Asterisks indicate significant differences between men and women. Substantially more patients of the validation cohort had a history of hypertension, atrial fibrillation and coronary artery disease in comparison to the derivation cohort; potentially reflecting veridical sampling differences or differences in data acquisition.

	**All participants (n=503)**	**Women (n=204)**	**Men (n=299)**	**Statistical comparison of male and**

				**female patients**
**Age**	65.0(14.6)	65.3(16.3)	64.8(13.2)	*p*=0.70
**Sex**	59% male,41% female	-	-	
**NIHSS**	5.5(5.4) (median(iqr): 4(5))	5.8(5.6) (median(iqr): 4(6))	5.3(5.2) (median(iqr): 4(5))	*p*=0.31
**Normalized DWI-derived stroke lesion volume (ml)**	21.0(40.5) (median(iqr): 3.9(19.8))	22.9(38.7) (median(iqr): 4.0(31.3))	19.7(41.7) (median(iqr): 3.7(15.0))	*p*=0.38
**White matter hyperintensity lesion volume (ml)**	11.13(13.6) (median(iqr): 5.8(12.3))	9.6(11.6) (median(iqr): 4.9(13.2))	12.2(14.8) (median(iqr): 6.2(12.4))	*p*=0.04*
**Hypertension**	63.6%	67.2%	61,2%	*p*=0.19
**Diabetes mellitus type 2**	22.7%	20.6%	24.1%	*p*=0.39
**Atrial fibrillation**	19.3%	24.0%	16.1%	*p*=0.03*
**Coronary artery disease**	16.9%	11.8%	20.4%	*p*=0.01*

Low-dimensional lesion embedding via non-negative matrix factorization

We once again estimated ten lesion atoms that represented typical voxel-wise lesion pattern. The derived lesion atoms could be matched with those atoms estimated for the data in the derivation cohort, which facilitated the comparison of results further (**Supplementary Table 3**).

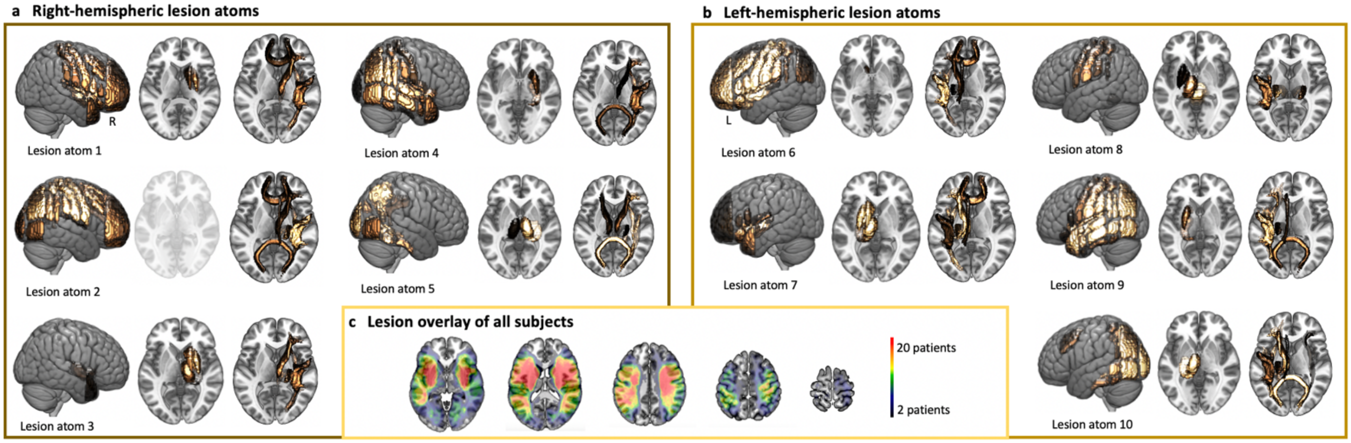

Supplementary Figure 3. Low-dimensional lesion representation in the validation cohort. a Right-hemispheric lesion atoms. b Left-hemispheric lesion atoms. c Lesion overlay. Source data are provided as a Source Data file.

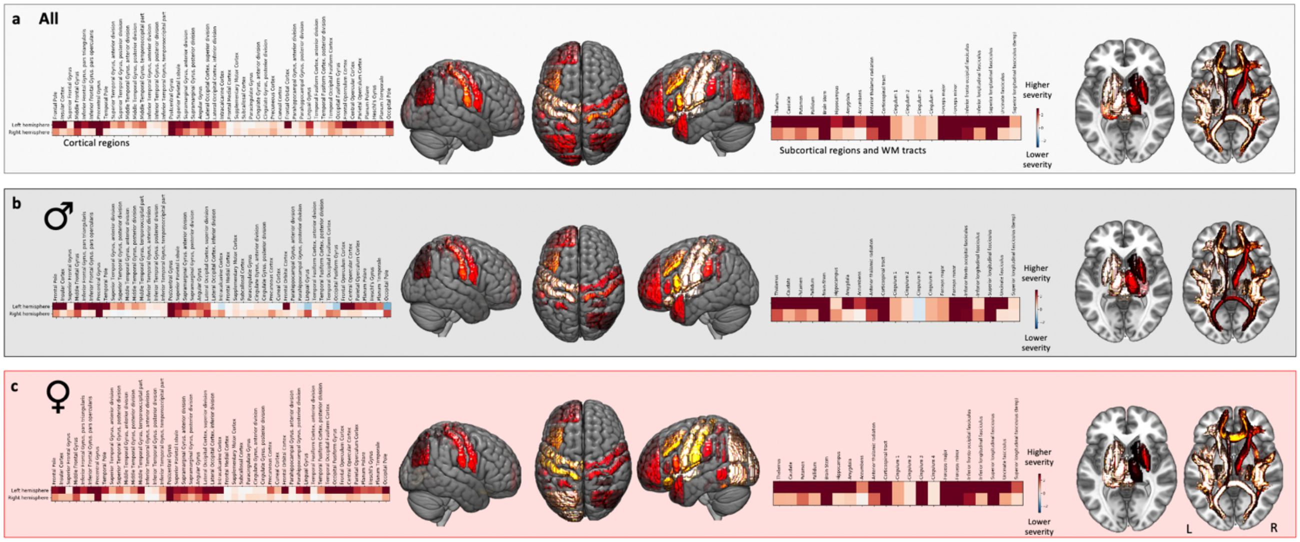

Supplementary Figure 4. Predictive relevances of individual brain regions for all (a), male (b) and female patients (c). Once again, female patients featured a more widespread pattern, particularly comprising brain areas in the left posterior circulation. Source data are provided as a Source Data file.

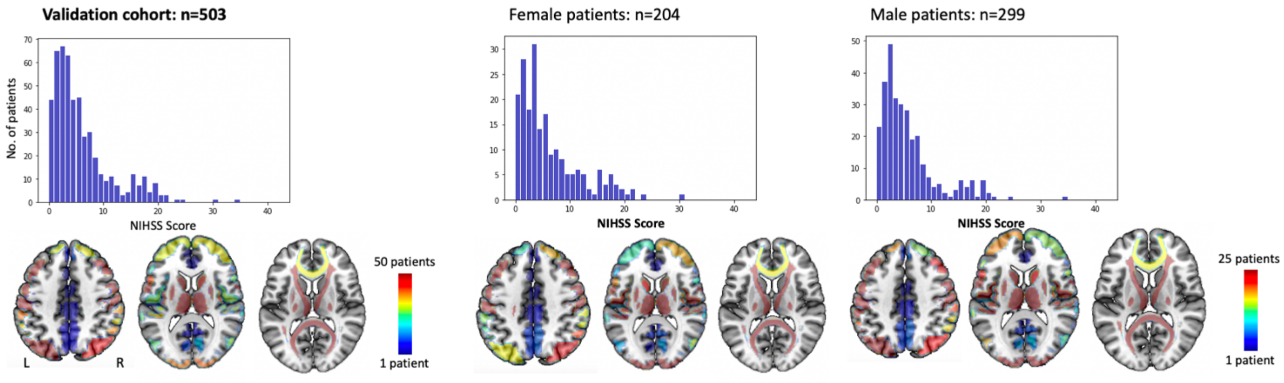

Supplementary Figure 5. NIHSS distributions and frequency maps for the entire validation cohort, as well as subgroups of only female and male patients. In case of either left- or right-hemispheric brain regions of interest, neither the frequencies of how often each brain region was affected, nor the region-wise lesion loads differed significantly between men and women (two-sided t-tests (lesion loads) and two-sided Fisher's exact tests (frequencies), $p>0.05$, Bonferroni-corrected for multiple comparisons). While the male- and female-specific lesion loads in the brainstem did not differ significantly, the brainstem was more frequently affected in male than in female patients (two-sided Fisher's exact test (frequencies), $p=0.03$, Bonferroni-corrected for multiple comparisons). Source data are provided as a Source Data file.

Supplementary note 3: Ancillary analyses: Data acquisition upon admission

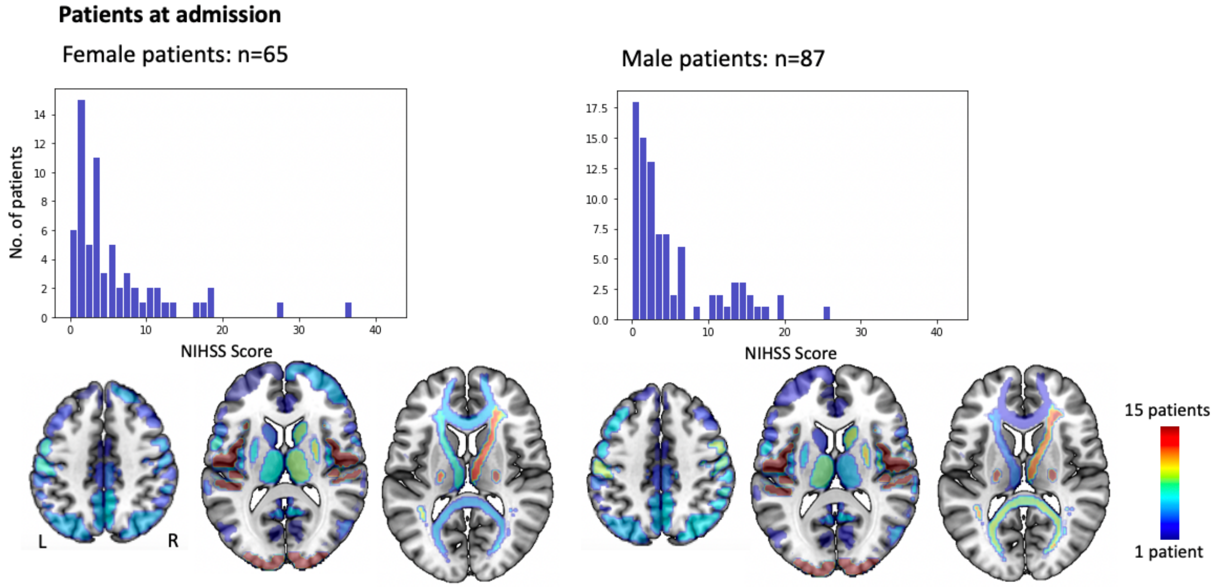

Supplementary Figure 6. NIHSS distributions and frequency maps for female and male patients with data acquisition upon admission. Neither the frequencies of how often each brain region was affected, nor the region-wise lesion loads differed significantly between men and women of the subgroup with more immediate data acquisition (two-sided t-tests (lesion loads) and two-sided Fisher's exact tests (frequencies), $p>0.05$, Bonferroni-corrected for multiple comparisons). Source data are provided as a Source Data file.

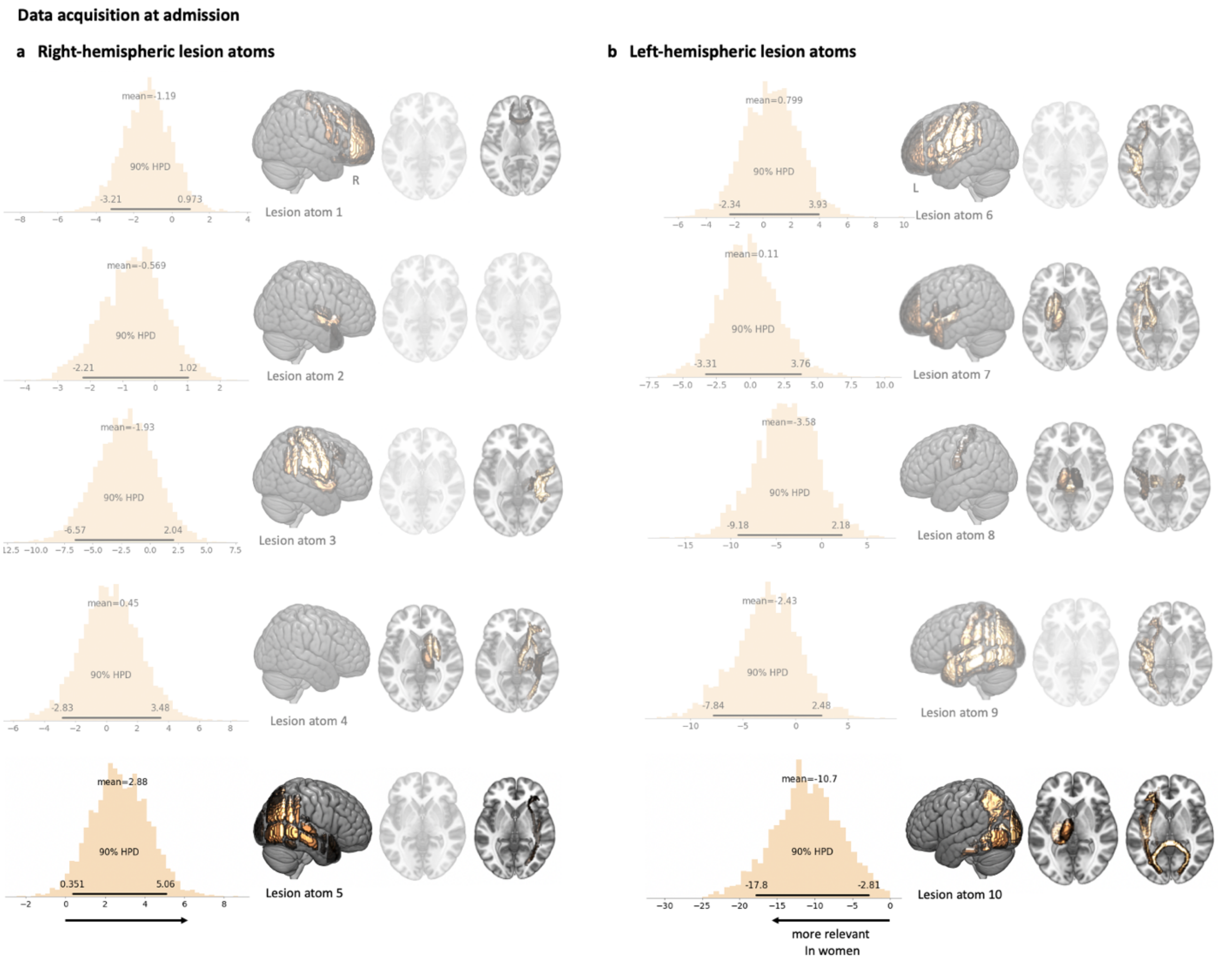

Supplementary Figure 7. Difference distributions of male- and female-specific Bayesian posteriors for all ten lesion atoms in the right (a) and left (b) hemisphere. Lesion atom 10 indicates a more pronounced effect on stroke severity in women. While lesion atom 5 suggests a more pronounced effect in men, this difference primarily originated from a negative lesion atom effect in women (i.e., lesions in these brain regions were associated with less severe strokes in women). Additionally, lesion atom 5 did not have any substantial relevance in men (i.e., the Bayesian posterior distribution overlapped with zero). Lesion atoms without any substantial sex differences are shown in transparent. Source data are provided as a Source Data file.

Supplementary note 4: Ancillary analyses: Stratifying for cardioembolic stroke genesis

Data of the derivation and validation cohorts were merged in ancillary analyses focused on the stroke subtype and age of participants. More specifically, we adopted the NMF-transformation as learned exclusively based on the derivation cohort and applied the same transformation to the validation cohort. To harmonize data of both cohorts further, we normalized NMF-transformed lesion data before merging and inserting it into the linear regression model. We refrained from re-computing the NMF-transformation based on the merged data and chose to rely on the derivations cohort's lesion embedding instead to increase the ease of interpretation: In this way, any results could immediately be compared to the derivation cohort's results. This choice was furthermore supported by our analyses demonstrating the independence of the concrete lesion embedding (c.f., **Results: Validation analyses**). Lastly, we added cohort membership as a covariate to the model to further account for differences between cohorts. The full model specification is given below:

Hyperpriors

$$hyper\_\sigma\_\beta \sim Halfcauchy(5)$$

$$\sigma\_\beta_{m,f} \sim Halfcauchy(hyper\_\sigma\_\beta)$$

$$hyper\_hyper\_mu\_\beta \sim Normal(\mu = 0, \sigma = 10)$$

$$hyper\_mu\_\beta_{ce,nonce} \sim Normal(\mu = hyper\_hyper\_mu\_\beta, \sigma = 10)$$

$$mu\_\beta_{ce,nonce * m,f} \sim Normal(\mu = hyper\_mu\_\beta_{ce,nonce}, \sigma = 10)$$

$$mu\_\alpha_{study} \sim Normal(\mu = 0, \sigma = 1)$$

Priors

$$\alpha \sim Normal(\mu = 0, \sigma = 1)$$

$$\alpha_{study} \sim Normal(\mu = mu\_\alpha_{study}, \sigma = 1)$$

$$\beta_{1\text{-}10;\ nonce,\ ce * m,f} \sim Normal(\mu = mu\_\beta_{ce,nonce * m,f}, \sigma = \sigma\_\beta_{m,f})$$

$$\beta_{age} \sim Normal(\mu = 0 , \sigma = 10)$$

$$\beta_{age*age} \sim Normal(\mu = 0 , \sigma = 10)$$

$$\beta_{sex} \sim Normal(\mu = 0 , \sigma = 1)$$

$$\beta_{hypertension} \sim Normal(\mu = 0 , \sigma = 1)$$

$$\beta_{diabetes} \sim Normal(\mu = 0 , \sigma = 1)$$

$$\beta_{atrial\ fibrillation} \sim Normal(\mu = 0 , \sigma = 1)$$

$$\beta_{coronary\ artery\ disease} \sim Normal(\mu = 0 , \sigma = 1)$$

$$\beta_{WMHv} \sim Normal(\mu = 0 , \sigma = 1)$$

Likelihood of linear model

$$NIHSS\_est = \alpha + \alpha_{study}[study] + \beta_{1\text{-}10}\ [(ce\ vs.\ non\text{-}ce) * (female\ vs.\ male)] * NMF\text{-}Component_{1\text{-}10} + \beta_{age} * Age\ \beta_{age*age} * Age^{2} + \beta_{sex} * Sex + \beta_{hypertension} * \text{hypertension} + \beta_{diabetes} * \text{diabetes} + \beta_{atrial\ fibrillation} * \text{atrial fibrillation} + \beta_{coronary\ artery\ disease} * \text{coronary artery disease} + \beta_{WMHv} * \text{WMHv}$$

Model likelihood

$$eps \sim Halfcauchy(20)$$

$$stroke\_severity \sim Normal(\mu = NIHSS\_est, \sigma = eps)$$

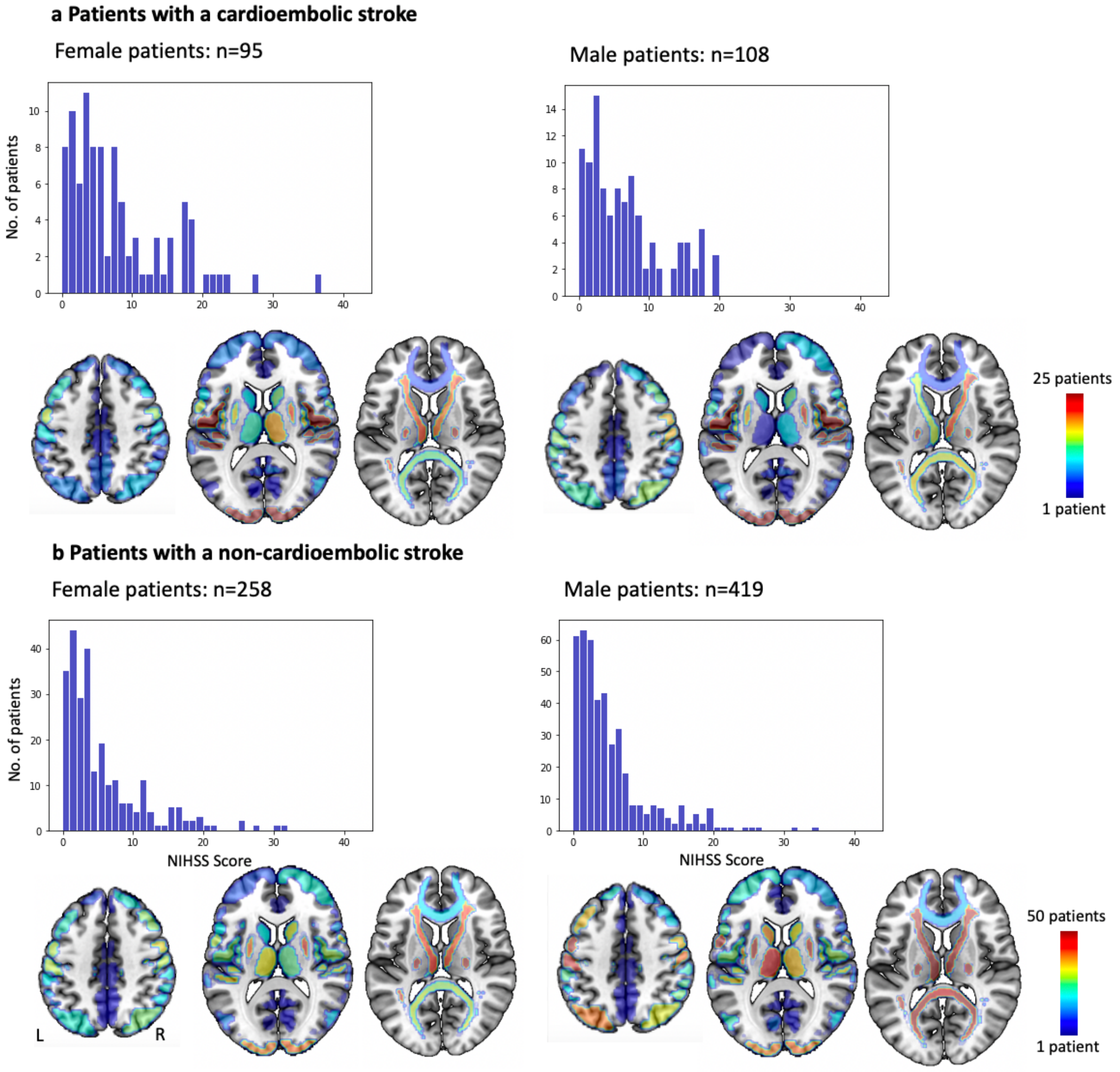

Supplementary Figure 8. NIHSS distributions and frequency maps for subgroups of female and male patients with (a) and without (b) cardioembolic stroke. Neither the frequencies of how often each brain region was affected, nor the region-wise lesion loads differed significantly between men and women of the cardioembolic and non-cardioembolic subgroups (two-sided t-tests (lesion loads) and two-sided Fisher's exact tests (frequencies), $p>0.05$, Bonferroni-corrected for multiple comparisons). Source data are provided as a Source Data file.

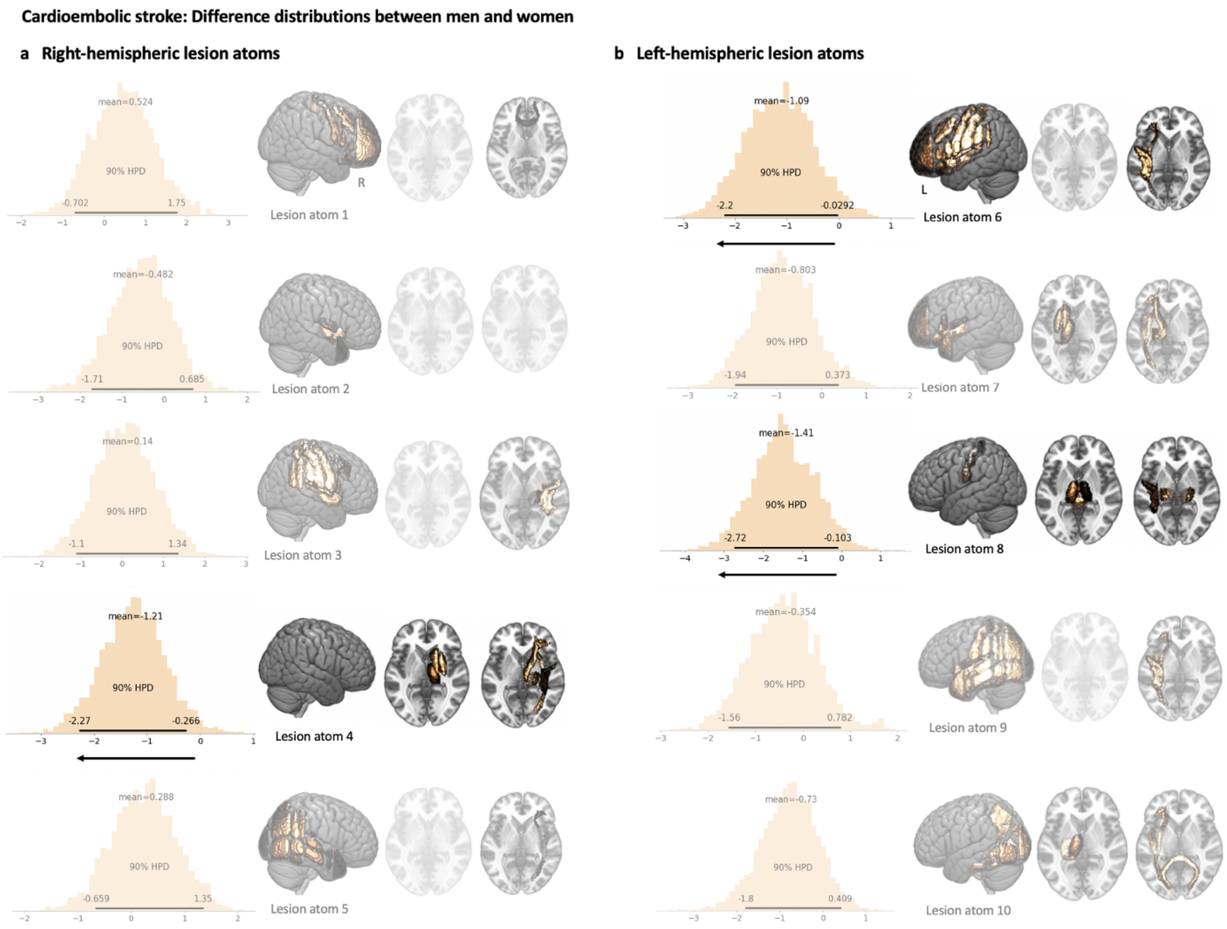

Supplementary Figure 9. Difference distributions of male- and female-specific Bayesian posteriors for all ten lesion atoms in the right (a) and left (b) hemisphere of all patients with a cardioembolic stroke genesis. Source data are provided as a Source Data file.

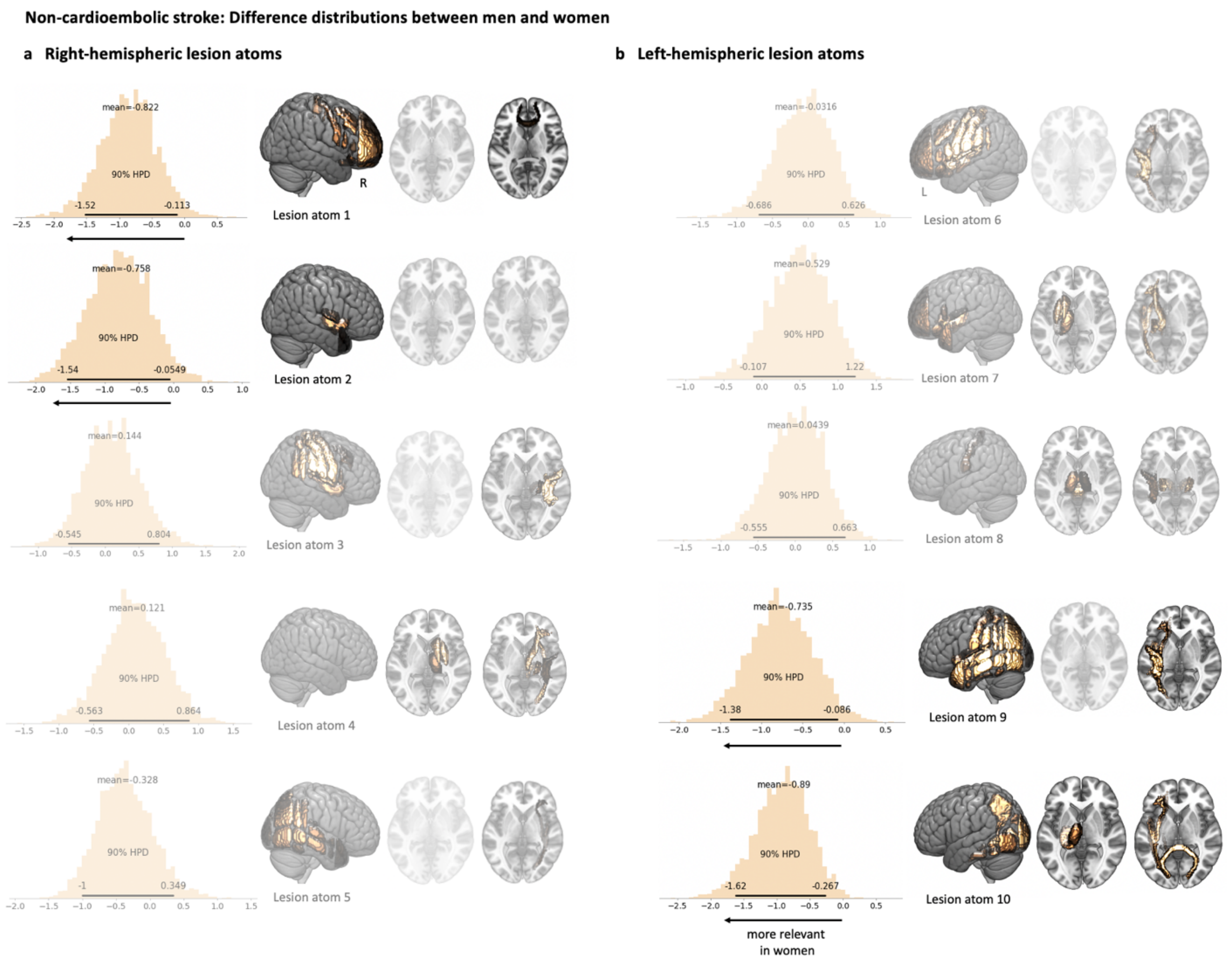

Supplementary Figure 10. Difference distributions of male- and female-specific Bayesian posteriors for all ten lesion atoms in the right (a) and left (b) hemisphere of all patients with a non-cardioembolic stroke genesis. Source data are provided as a Source Data file.

Supplementary note 5: Ancillary analyses: Stratifying for median age at menopause

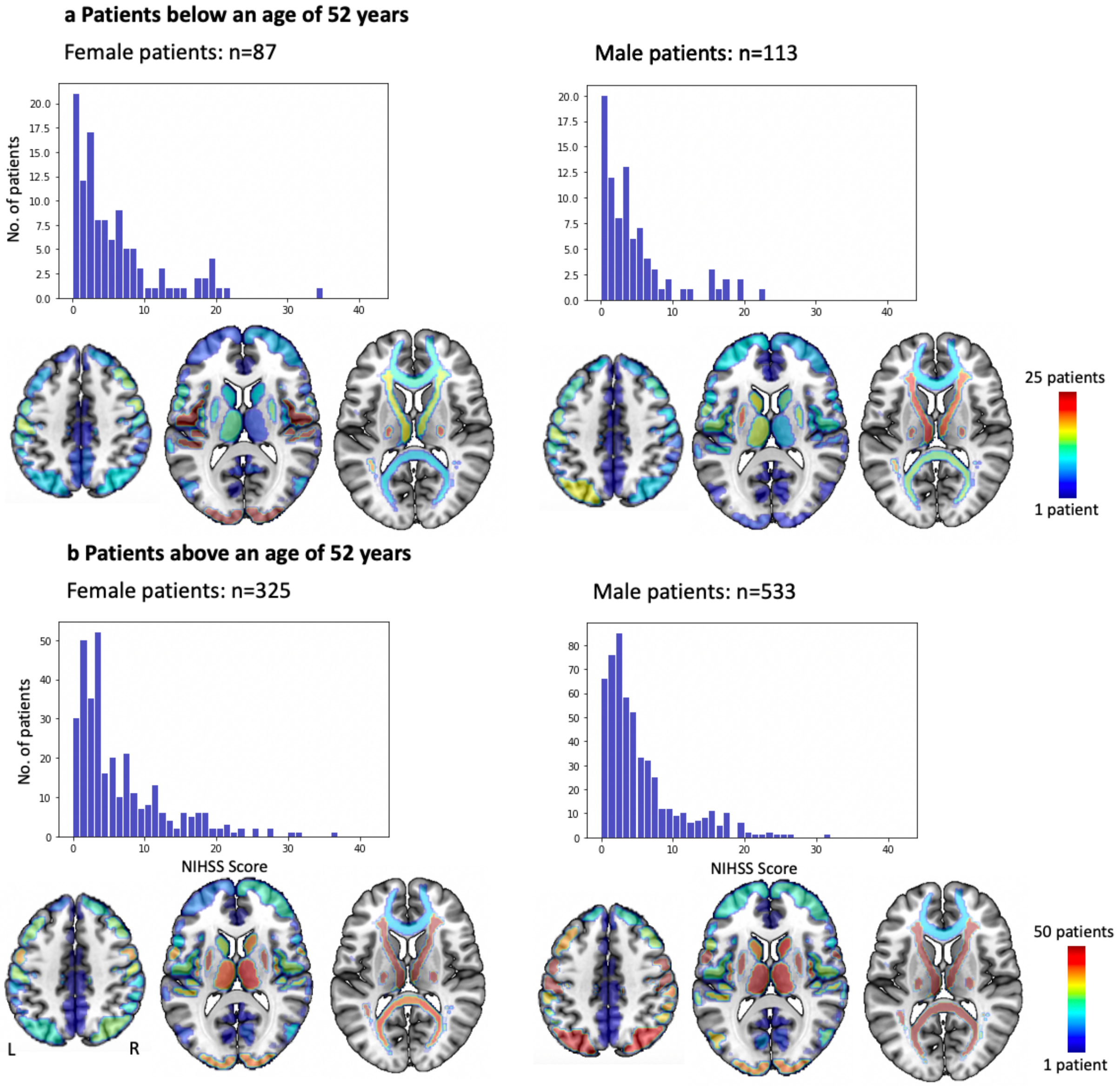

Supplementary Figure 11. NIHSS distributions and frequency maps for subgroups of female and male patients below (a) and above (b) 52 years, i.e., the median age at menopause. Neither the frequencies of how often each brain region was affected, nor the region-wise lesion loads differed significantly between men and women of the two age subgroups (two-sided t-tests (lesion loads) and two-sided Fisher's exact tests (frequencies), $p>0.05$, Bonferroni-corrected for multiple comparisons). Source data are provided as a Source Data file.

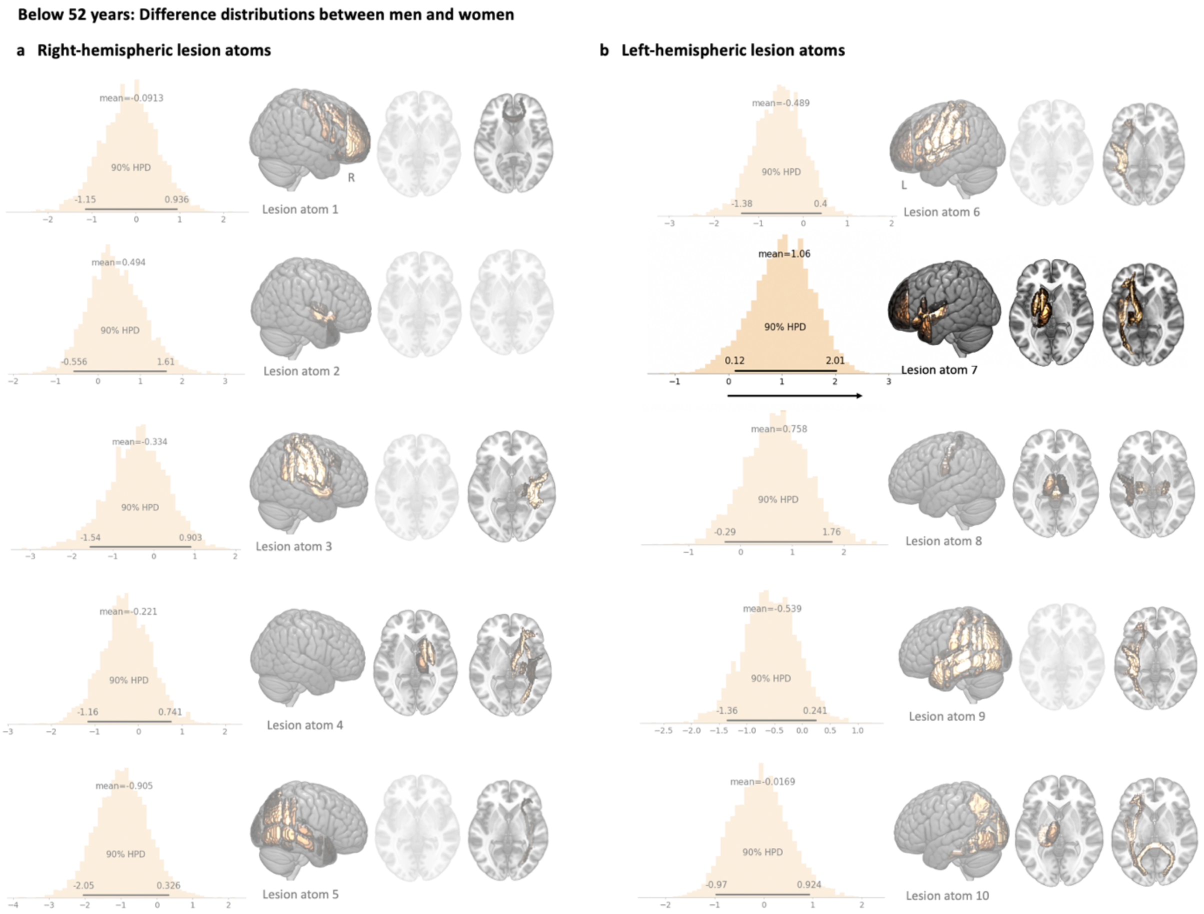

Supplementary Figure 12. Difference distributions of male- and female-specific Bayesian posteriors for all ten lesion atoms in the right (a) and left (b) hemisphere of all patients with an age below 52 years. Source data are provided as a Source Data file.

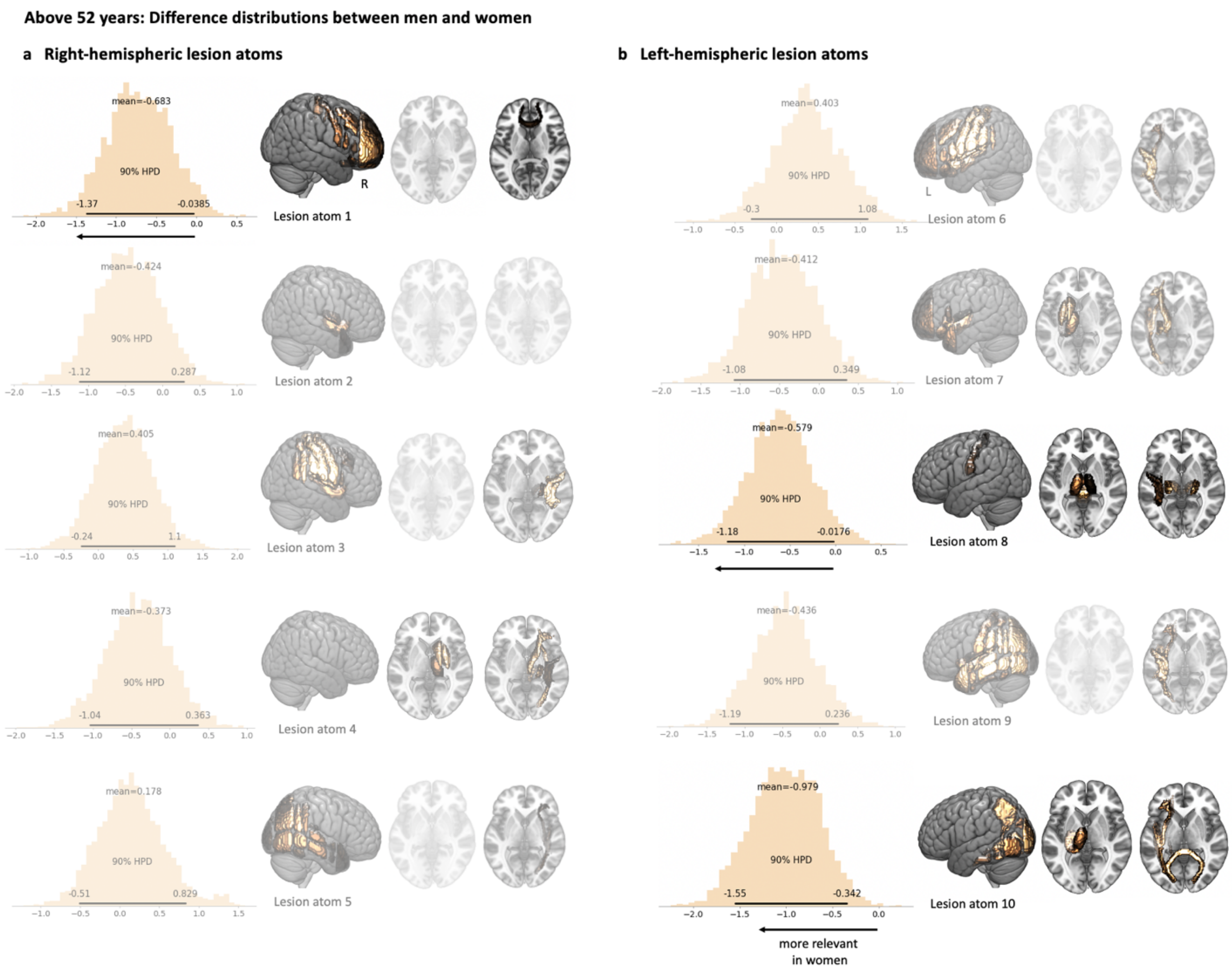

Supplementary Figure 13. Difference distributions of male- and female-specific Bayesian posteriors for all ten lesion atoms in the right (a) and left (b) hemisphere of all patients with an age above 52 years. Source data are provided as a Source Data file.

Supplementary Table 3. Matching of lesion atoms via Pearson correlations.

**Derivation cohort: Number of lesion atom**	**Validation cohort: Number of lesion atom**	**Pearson correlation of NMF-weights** (uncorrected p-values)
1	1	r=0.54, p=3.1e^{-11}
2	1	r=0.67, p=7.4e^{-18}
3	2	r=0.69, p=8.9e^{-20}
4	3	r=0.89, p=1.3e^{-44}
5	4 (5)	r=0.85, p=1.2e^{-37}

		(r=0.17, p=5.1e^{-2})
6	6	r=0.84, p=1.0e^{-35}
7	7	r=0.96, p=5.1e^{-69}
8	8	r=0.95, p=2.2e^{-63}
9	9	r=0.91, p=1.1e^{-50}
10	10	r=0.92, p=2.4e^{-52}

Supplementary References

1. Lee, D. D. & Seung, H. S. Learning the parts of objects by non-negative matrix factorization. *Nature* **401**, 788 (1999).
2. Giese, A.-K. *et al.* Design and rationale for examining neuroimaging genetics in ischemic stroke: The MRI-GENIE study. *Neurology Genetics* **3**, e180 (2017).
3. Wu, O. *et al.* Big Data Approaches to Phenotyping Acute Ischemic Stroke Using Automated Lesion Segmentation of Multi-Center Magnetic Resonance Imaging Data. *Stroke* STROKEAHA. 119.025373 (2019).
4. Wu, O. A multi-center investigation of the association of acute stroke severity and long-term outcome with acute stroke lesion topography. in *JOURNAL OF CEREBRAL BLOOD FLOW AND METABOLISM* vol. 39 38–38 (SAGE PUBLICATIONS INC 2455 TELLER RD, THOUSAND OAKS, CA 91320 USA, 2019).
5. Schirmer, M. D. *et al.* White matter hyperintensity quantification in large-scale clinical acute ischemic stroke cohorts–The MRI-GENIE study. *NeuroImage: Clinical* 101884 (2019).
