# Supplementary material for: Outcome after acute ischemic stroke is linked to sex-specific lesion patterns
Source: Nat Commun. 2021 Jun 2;12:3289. doi: 10.1038/s41467-021-23492-3 (PMC8172535; doi:10.1038/s41467-021-23492-3)
Supplement: Supplementary file 3 — Descriptions of Additional Supplementary Files [file 41467_2021_23492_MOESM3_ESM.pdf]

Descriptions of Additional Supplementary Files

Supplementary Data 1

Description: Statistical comparisons of region-wise lesion loads and frequencies between men and women in the derivation cohort. Numbers in women and men were compared via two-sided t-tests (lesion loads) and two-sided Fisher's exact tests (frequencies). We here present resulting p-values, corrected for multiple comparisons.

Supplementary Data 2

Description: Statistical comparisons of region-wise lesion loads and frequencies between the left in the derivation cohort**.** Numbers in the left versus right hemisphere were compared via two-sided t-tests (lesion loads) and two-sided Fisher's exact tests (frequencies). We here present resulting p-values, corrected for multiple comparisons.
