# Supplementary material for: Outcome after acute ischemic stroke is linked to sex-specific lesion patterns
Source: Nat Commun. 2021 Jun 2;12:3289. doi: 10.1038/s41467-021-23492-3 (PMC8172535; doi:10.1038/s41467-021-23492-3)
Supplement: Supplementary file 6 — Reporting Summary [file 41467_2021_23492_MOESM6_ESM.pdf]

nature research

Corresponding author(s): Bonkhoff, A.K.

Last updated by author(s): Apr 15, 2021

Reporting Summary

Nature Research wishes to improve the reproducibility of the work that we publish. This form provides structure for consistency and transparency in reporting. For further information on Nature Research policies, see our Editorial Policies and the Editorial Policy Checklist.

Statistics

For all statistical analyses, confirm that the following items are present in the figure legend, table legend, main text, or Methods section.

n/a	Confirmed	
☐	☒	The exact sample size (*n*) for each experimental group/condition, given as a discrete number and unit of measurement
☐	☒	A statement on whether measurements were taken from distinct samples or whether the same sample was measured repeatedly
☐	☒	The statistical test(s) used AND whether they are one- or two-sided<br>*Only common tests should be described solely by name; describe more complex techniques in the Methods section.*
☐	☒	A description of all covariates tested
☐	☒	A description of any assumptions or corrections, such as tests of normality and adjustment for multiple comparisons
☐	☒	A full description of the statistical parameters including central tendency (e.g. means) or other basic estimates (e.g. regression coefficient) AND variation (e.g. standard deviation) or associated estimates of uncertainty (e.g. confidence intervals)
☐	☒	For null hypothesis testing, the test statistic (e.g. *F*, *t*, *r*) with confidence intervals, effect sizes, degrees of freedom and *P* value noted<br>*Give P values as exact values whenever suitable.*
☐	☒	For Bayesian analysis, information on the choice of priors and Markov chain Monte Carlo settings
☐	☒	For hierarchical and complex designs, identification of the appropriate level for tests and full reporting of outcomes
☐	☒	Estimates of effect sizes (e.g. Cohen's *d*, Pearson's *r*), indicating how they were calculated

Our web collection on statistics for biologists contains articles on many of the points above.

Software and code

Policy information about availability of computer code

Data collection	No specific software was used for data collection.
Data analysis	Preprocessing of structural MRI images was conducted in MRIcron (versions 04/2010, 08/2014, 2019) and Matlab 2019b, particularly relying on the toolbox SPM (version SPM 12). Further statistical evaluations, esp. Bayesian analyses, were conducted in python 3.7 (packages pymc3 and nilearn). The code has been deposited openly available here: https://github.com/AnnaBonkhoff/BMH_stroke_severity_sex_differences (Bonkhoff AK et al. Outcome after acute ischemic stroke is linked to sex-specific lesion patterns. http://doi.org/10.5281/zenodo.4670275. Zenodo (2021).)

For manuscripts utilizing custom algorithms or software that are central to the research but not yet described in published literature, software must be made available to editors and reviewers. We strongly encourage code deposition in a community repository (e.g. GitHub). See the Nature Research guidelines for submitting code & software for further information.

Data

Policy information about availability of data

All manuscripts must include a data availability statement. This statement should provide the following information, where applicable:

- Accession codes, unique identifiers, or web links for publicly available datasets
- A list of figures that have associated raw data
- A description of any restrictions on data availability

The authors agree to make the data of development and validation cohorts available to any researcher for the express purposes of reproducing the here presented results and with the explicit permission for data sharing by Massachusetts General Hospital's institutional review board. The Harvard-Oxford and JHU DTI-based white matter atlases are accessible online (https://fsl.fmrib.ox.ac.uk/fsl/fslwiki/Atlases). Source data for all figures are provided with this paper.

Field-specific reporting

Please select the one below that is the best fit for your research. If you are not sure, read the appropriate sections before making your selection.

☒ Life sciences ☐ Behavioural & social sciences ☐ Ecological, evolutionary & environmental sciences

For a reference copy of the document with all sections, see nature.com/documents/nr-reporting-summary-flat.pdf

Life sciences study design

All studies must disclose on these points even when the disclosure is negative.

Sample size	We had access to 668 patients with manual lesion segmentations. Quality control of normalization results of structural images led to the exclusion of 55 out of these 668 patients. Included and excluded patients did not differ significantly with respect to age, sex and stroke severity (mean age(SD): 65.0(15.1) vs. 64.0(14.8), p=0.66, sex: 38% female vs. 35% female, p=0.77, mean NIHSS(SD): 5.04(6.0) vs. 6.1(6.0), p=0.24). 555 out of the 613 remaining patients had complete data with respect to clinical variables (i.e., age, sex, stroke severity, comorbidities, WMH lesion volume) and were thus included in final analyses.
Data exclusions	We had pre-established criteria for subject exclusion to ensure a high quality of included data and furthermore allow the correction for important clinical covariates. These exclusion criteria were: incomplete imaging or clinical data and insufficient quality of preprocessed lesion outlines. In this study, 55 patients were excluded due to insufficient quality of their normalized structural images, 48 of the remaining patients with usable scans were excluded due to missing clinical information on age, sex, stroke severity, comorbidities or WMH lesion volume.
Replication	We repeated analyses in an independent dataset of 503 ischemic stroke patients (age: 65.0 (14.6), sex: 40.6% female, mean NIHSS: 5.48 (5.35)), acquired within the framework of the multi-site MRI-GENIE study, to test the robustness of our findings. We could here validate our preprocessing pipeline, i.e., derived low-dimensional lesion representations were highly correlated between the datasets. Furthermore, region-wise main relevances when explaining stroke severity across men and women were highly similar (main relevances: subcortically in the left and right hemisphere as well as in bilateral pre- and postcentral gyri and left-hemispheric insular and opercular regions). When repeating sex-specific analyses, women once again presented more widespread lesion pattern of stroke severity. In particular, one lesion atom representing left posterior circulation brain regions was substantially more relevant in women. No further replication attempts were conducted.
Randomization	N/A (observational studies)
Blinding	N/A (observational studies)

Reporting for specific materials, systems and methods

We require information from authors about some types of materials, experimental systems and methods used in many studies. Here, indicate whether each material, system or method listed is relevant to your study. If you are not sure if a list item applies to your research, read the appropriate section before selecting a response.

Materials & experimental systems

n/a	Involved in the study
☒	☐ Antibodies
☒	☐ Eukaryotic cell lines
☒	☐ Palaeontology and archaeology
☒	☐ Animals and other organisms
☐	☒ Human research participants
☒	☐ Clinical data
☒	☐ Dual use research of concern

Methods

n/a	Involved in the study
☒	☐ ChIP-seq
☒	☐ Flow cytometry
☐	☒ MRI-based neuroimaging

Human research participants

Policy information about studies involving human research participants

Population characteristics	Acute ischemic stroke (AIS) patients (n=555), considered as development cohort in this study, were admitted to Massachusetts General Hospital and enrolled as of part the Genes Associated with Stroke Risk and Outcomes Study (GASROS; mean age (standard deviation (SD)): 65.0 (14.8) years, 38% females).
Recruitment	Inclusion was generally considered for any AIS patient that met the following criteria; i) adult patients ≥18 years of age, ii) admitted to the emergency department with signs and symptoms of AIS, and iii) neuroimaging confirmation of an acute infarct. Only subjects with MRI data obtained within 48 hours from stroke onset as well as complete phenotypic data, such as stroke severity and stroke risk factors were included in this study (i.e., complete case analyses). We furthermore excluded patients with insufficient scan quality. Conceivably, particularly severely affected patients may move more during scan acquisition, leading to a poorer scan quality and higher likelihood of exclusion. We could, however, not find any significant differences for the age, sex and NIHSS of included and excluded patients.
Ethics oversight	Institutional Review Protocols Massachusetts General Hospital #: 2001P001186 and #: 2003P000836. All subjects gave written informed consent in accordance with the Declaration of Helsinki.

Note that full information on the approval of the study protocol must also be provided in the manuscript.

Magnetic resonance imaging

Experimental design

Design type	Lesion symptom mapping analysis
Design specifications	one measurement time point per patient (~10 mins of scanning time, in the first 48 hours after admission)
Behavioral performance measures	National Institutes of Health Stroke Scale (NIHSS)-based Stroke severity

Acquisition

Imaging type(s)	Structural MRI
Field strength	1.5T General Electric Signa scanner (very few cases on 1.5 or 3T Siemens scanner)
Sequence & imaging parameters	Axial T2 FLAIR images: TR 5,000 ms, minimum TE of 62 to 116 ms, TI 2,200 ms, FOV 220–240 mm
Area of acquisition	Whole-brain scans

Diffusion MRI ☒ Used ☐ Not used

Parameters: DWI: repetition time (TR) 5,000 ms, minimum echo time (TE) of 62 to 117 ms, field-of-view (FOV) 220 mm field-of-view, 5-mm slice thickness with a 1-mm gap, and 0 s/mm2 (b-zero) and 1000 s/mm2 b-values

Preprocessing

Preprocessing software	SPM (SPM12; http://www.fil.ion.ucl.ac.uk/spm/) in Matlab 2019b (The Mathworks, Natick, MA, USA)
Normalization	1. Linear realignment of both DWI and FLAIR images with MNI template (TPM.nii)<br>2. Co-registration of DWI image to the FLAIR image<br>3. Denoising of the images (code: https://github.com/brudfors/spm_superres)<br>4. Non-linear normalization of FLAIR image (with masked lesions, i.e. the lesioned tissue was set to zero during normalization to decrease the likelihood of spatial distortions)
Normalization template	Montreal Neurological Institute (MNI-152) template: TPM.nii (as included in SPM toolbox)
Noise and artifact removal	c.f., step 3 under normalization: We ran a model-based denoising algorithm for multi-sequence MR images that fitted a least-squares fidelity term and a joint total variation (JTV) regularisation term. The hyper-parameters were estimated from the data. This step was performed to reduce noise artifacts in the images to improve the spatial normalization.
Volume censoring	N/A

Statistical modeling & inference

Model type and settings	Bayesian (hierarchical) linear regression models with broad, normal priors
Effect(s) tested	Main effects: 10 (male- and female-specific) lesion patterns

Specify type of analysis: ☒ Whole brain ☐ ROI-based ☐ Both

Statistic type for inference
(See Eklund et al. 2016)

Lesion-pattern-wise (all patterns were tested within the same model)

Correction

N/A (just one whole-brain model)

Models & analysis

n/a	Involved in the study
☒	☐ Functional and/or effective connectivity
☒	☐ Graph analysis
☐	☒ Multivariate modeling or predictive analysis

Multivariate modeling and predictive analysis

We employed Bayesian hierarchical linear regression to perform multivariate lesion symptom mapping. Input variables were 10 lesion atoms, age, age2, sex, hypertension, diabetes, atrial fibrillation, coronary artery disease, WMH volume (Normal priors with mean of 0). The outcome variable was NIHSS-based stroke severity. We used the No U-Turn Sampler (NUTS), a type of Monte Carlo Markov Chain algorithm (setting: draws=5000) to draw samples from the Bayesian posterior parameter distributions that were eventually interpreted.
